# Supplementary material for: Multi‐dataset identification of innovative feature genes and molecular mechanisms in keratoconus
Source: J Cell Mol Med. 2024 Sep 19;28(18):e70079. doi: 10.1111/jcmm.70079 (PMC11412914; doi:10.1111/jcmm.70079)
Supplement: Supplementary file 2 — Material S2. [file JCMM-28-e70079-s002.zip › 7.GSEA analysis/3.DBNDD1/KEGG_CARDIAC_MUSCLE_CONTRACTION.html]

Details for gene set KEGG\_CARDIAC\_MUSCLE\_CONTRACTION[GSEA]

|  || Dataset | DBNDD1.group.cls#h\_versus\_l |
| Phenotype | group.cls#h\_versus\_l |
| Upregulated in class | l |
| GeneSet | KEGG\_CARDIAC\_MUSCLE\_CONTRACTION |
| Enrichment Score (ES) | -0.31247056 |
| Normalized Enrichment Score (NES) | -1.1663946 |
| Nominal p-value | 0.17175573 |
| FDR q-value | 0.68473977 |
| FWER p-Value | 0.993 |
Table: GSEA Results Summary

  

Fig 1: Enrichment plot: KEGG\_CARDIAC\_MUSCLE\_CONTRACTION      
 Profile of the Running ES Score & Positions of GeneSet Members on the Rank Ordered List

  

| SYMBOL | TITLE | RANK IN GENE LIST | RANK METRIC SCORE | RUNNING ES | CORE ENRICHMENT || 1 | TNNT2 | na | 514 | 0.448 | 0.0341 | No |
| 2 | CACNB1 | na | 587 | 0.431 | 0.0791 | No |
| 3 | TNNI3 | na | 657 | 0.420 | 0.1229 | No |
| 4 | CACNA1F | na | 1766 | 0.316 | 0.1254 | No |
| 5 | ACTC1 | na | 2612 | 0.267 | 0.1302 | No |
| 6 | ATP1B4 | na | 2619 | 0.267 | 0.1592 | No |
| 7 | CACNG5 | na | 2873 | 0.257 | 0.1799 | No |
| 8 | CACNG7 | na | 4144 | 0.209 | 0.1661 | No |
| 9 | TPM2 | na | 5034 | 0.180 | 0.1600 | No |
| 10 | TNNC1 | na | 7636 | 0.111 | 0.0971 | No |
| 11 | CACNG1 | na | 8316 | 0.098 | 0.0882 | No |
| 12 | COX7A2L | na | 8857 | 0.088 | 0.0822 | No |
| 13 | COX6A2 | na | 8938 | 0.087 | 0.0894 | No |
| 14 | COX4I2 | na | 9035 | 0.085 | 0.0959 | No |
| 15 | ATP1B1 | na | 9417 | 0.080 | 0.0936 | No |
| 16 | CACNA2D3 | na | 10208 | 0.068 | 0.0783 | No |
| 17 | COX6B2 | na | 10294 | 0.067 | 0.0832 | No |
| 18 | ATP2A2 | na | 11124 | 0.057 | 0.0654 | No |
| 19 | ATP1A3 | na | 11874 | 0.048 | 0.0491 | No |
| 20 | CACNA1D | na | 13311 | 0.036 | 0.0116 | No |
| 21 | UQCR11 | na | 13449 | 0.035 | 0.0114 | No |
| 22 | COX8A | na | 13890 | 0.031 | 0.0021 | No |
| 23 | CACNB2 | na | 14012 | 0.030 | 0.0018 | No |
| 24 | TPM4 | na | 14283 | 0.028 | -0.0029 | No |
| 25 | COX6B1 | na | 15292 | 0.021 | -0.0298 | No |
| 26 | COX5B | na | 15394 | 0.020 | -0.0305 | No |
| 27 | CACNG8 | na | 15428 | 0.020 | -0.0293 | No |
| 28 | COX7B2 | na | 15452 | 0.020 | -0.0278 | No |
| 29 | COX7B | na | 15536 | 0.019 | -0.0281 | No |
| 30 | COX7A2 | na | 15716 | 0.018 | -0.0314 | No |
| 31 | UQCR10 | na | 16010 | 0.016 | -0.0381 | No |
| 32 | UQCRC1 | na | 16639 | 0.012 | -0.0549 | No |
| 33 | UQCRC2 | na | 16785 | 0.011 | -0.0580 | No |
| 34 | COX4I1 | na | 16804 | 0.011 | -0.0573 | No |
| 35 | COX7C | na | 17085 | 0.009 | -0.0644 | No |
| 36 | UQCRQ | na | 17160 | 0.008 | -0.0657 | No |
| 37 | COX6C | na | 17183 | 0.008 | -0.0654 | No |
| 38 | COX5A | na | 17706 | 0.005 | -0.0799 | No |
| 39 | CYC1 | na | 17708 | 0.005 | -0.0794 | No |
| 40 | TPM1 | na | 17928 | 0.003 | -0.0854 | No |
| 41 | UQCRH | na | 18129 | 0.002 | -0.0909 | No |
| 42 | COX6A1 | na | 18979 | -0.004 | -0.1150 | No |
| 43 | SLC9A6 | na | 19070 | -0.005 | -0.1170 | No |
| 44 | UQCRFS1 | na | 19283 | -0.007 | -0.1224 | No |
| 45 | UQCRB | na | 19342 | -0.007 | -0.1233 | No |
| 46 | ATP1A4 | na | 21341 | -0.025 | -0.1782 | No |
| 47 | COX7A1 | na | 21665 | -0.029 | -0.1844 | No |
| 48 | TPM3 | na | 23026 | -0.043 | -0.2189 | No |
| 49 | CACNA1C | na | 23124 | -0.045 | -0.2168 | No |
| 50 | MYH7 | na | 23841 | -0.054 | -0.2316 | No |
| 51 | ATP1B3 | na | 25535 | -0.079 | -0.2718 | No |
| 52 | MYL2 | na | 25552 | -0.079 | -0.2636 | No |
| 53 | MYL3 | na | 25616 | -0.081 | -0.2566 | No |
| 54 | ATP1A1 | na | 25979 | -0.087 | -0.2575 | No |
| 55 | ATP1B2 | na | 26095 | -0.089 | -0.2511 | No |
| 56 | COX8C | na | 27951 | -0.130 | -0.2904 | No |
| 57 | CACNG4 | na | 28414 | -0.141 | -0.2884 | No |
| 58 | CACNB3 | na | 29250 | -0.164 | -0.2945 | Yes |
| 59 | CACNA2D4 | na | 29251 | -0.164 | -0.2766 | Yes |
| 60 | MYH6 | na | 29406 | -0.169 | -0.2626 | Yes |
| 61 | FXYD2 | na | 29524 | -0.173 | -0.2471 | Yes |
| 62 | ATP1A2 | na | 30814 | -0.214 | -0.2609 | Yes |
| 63 | CACNG6 | na | 31154 | -0.226 | -0.2459 | Yes |
| 64 | SLC9A1 | na | 31268 | -0.231 | -0.2239 | Yes |
| 65 | SLC8A1 | na | 32046 | -0.266 | -0.2173 | Yes |
| 66 | RYR2 | na | 32172 | -0.274 | -0.1910 | Yes |
| 67 | CACNA2D2 | na | 32648 | -0.304 | -0.1715 | Yes |
| 68 | CACNG2 | na | 33176 | -0.343 | -0.1493 | Yes |
| 69 | CACNA2D1 | na | 33832 | -0.408 | -0.1237 | Yes |
| 70 | CACNB4 | na | 33927 | -0.420 | -0.0806 | Yes |
| 71 | CACNA1S | na | 34256 | -0.474 | -0.0383 | Yes |
| 72 | CACNG3 | na | 34262 | -0.475 | 0.0134 | Yes |
Table: GSEA details [plain text format]

  

Fig 2: KEGG\_CARDIAC\_MUSCLE\_CONTRACTION      
 Blue-Pink O' Gram in the Space of the Analyzed GeneSet

  

Fig 3: KEGG\_CARDIAC\_MUSCLE\_CONTRACTION: Random ES distribution      
 Gene set null distribution of ES for **KEGG\_CARDIAC\_MUSCLE\_CONTRACTION**
